# Malaria rapid diagnostic kits: quality of packaging, design and labelling of boxes and components and readability and accuracy of information inserts

**DOI:** 10.1186/1475-2875-10-39

**Published:** 2011-02-13

**Authors:** Philippe Gillet, Jessica Maltha, Veerle Hermans, Raffaella Ravinetto, Cathrien Bruggeman, Jan Jacobs

**Affiliations:** 1 Department of Clinical Sciences, Institute of Tropical Medicine (ITM), Unit of Tropical Laboratory Medicine, Nationalestraat 155, B 2000 Antwerp, Belgium; 2 Faculty of Health, Medicine and Life Sciences (FHML), Maastricht, The Netherlands; 3 Clinical Trials Unit, Institute of Tropical Medicine (ITM), Antwerp, Belgium; 4 Department of Medical Microbiology, School for Public Health and Primary Care: CAPHRI, Maastricht University Medical Center, The Netherlands

## Abstract

**Background:**

The present study assessed malaria RDT kits for adequate and correct packaging, design and labelling of boxes and components. Information inserts were studied for readability and accuracy of information.

**Methods:**

Criteria for packaging, design, labelling and information were compiled from Directive 98/79 of the European Community (EC), relevant World Health Organization (WHO) documents and studies on end-users' performance of RDTs. Typography and readability level (Flesch-Kincaid grade level) were assessed.

**Results:**

Forty-two RDT kits from 22 manufacturers were assessed, 35 of which had evidence of good manufacturing practice according to available information (*i.e*. CE-label affixed or inclusion in the WHO list of ISO13485:2003 certified manufacturers). Shortcomings in devices were (i) insufficient place for writing sample identification (n = 40) and (ii) ambiguous labelling of the reading window (n = 6). Buffer vial labels were lacking essential information (n = 24) or were of poor quality (n = 16). Information inserts had elevated readability levels (median Flesch Kincaid grade 8.9, range 7.1 - 12.9) and user-unfriendly typography (median font size 8, range 5 - 10). Inadequacies included (i) no referral to biosafety (n = 18), (ii) critical differences between depicted and real devices (n = 8), (iii) figures with unrealistic colours (n = 4), (iv) incomplete information about RDT line interpretations (n = 31) and no data on test characteristics (n = 8). Other problems included (i) kit names that referred to *Plasmodium vivax *although targeting a pan-species *Plasmodium *antigen (n = 4), (ii) not stating the identity of the pan-species antigen (n = 2) and (iii) slight but numerous differences in names displayed on boxes, device packages and information inserts. Three CE labelled RDT kits produced outside the EC had no authorized representative affixed and the shape and relative dimensions of the CE symbol affixed did not comply with the Directive 98/79/EC. Overall, RDTs with evidence of GMP scored better compared to those without but inadequacies were observed in both groups.

**Conclusion:**

Overall, malaria RDTs showed shortcomings in quality of construction, design and labelling of boxes, device packages, devices and buffers. Information inserts were difficult to read and lacked relevant information.

## Background

### The use of malaria RDTs is rapidly expanding

Prompt parasitological confirmation by microscopy or alternatively by RDTs is recommended in all patients suspected of malaria before treatment is started [1]. As a consequence, malaria rapid diagnostic tests (RDTs) are increasingly used as a diagnostic tool in both malaria endemic and non-endemic settings: in 2007, more than 70,000,000 tests were performed [2].

Malaria RDTs are so-called immunochromatographic tests that detect *Plasmodium *antigens in the blood by an antigen-antibody reaction on a nitrocellulose strip. The antigen-antibody complex is conjugated to colloidal gold, and a positive result is visible as a cherry- or purple-red coloured line. Apart from a control line, there are one, two or three test lines: the so-called two-band tests comprise a control line and a single test line, and are mostly designed to diagnose *Plasmodium falciparum*. Their targets are either histidine-rich protein-2 (HRP-2) or *P. falciparum*-specific parasite lactate dehydrogenase (Pf-pLDH). Three-band RDTs display a second test line mostly targeting antigens common to the four species such as pan-*Plasmodium*-specific parasite lactate dehydrogenase (pan-pLDH) or aldolase. The four-band RDTs have an additional third test line targeting *Plasmodium vivax*-specific pLDH (Pv-pLDH).

### Written instructions add to the correct performance and interpretation of RDTs

RDTs are accurate and robust but they have limitations linked to design, production and distribution [3-8]. In addition, there are errors at the level of the end-user, which apply to both laboratory staff and field workers and are related to sampling, testing and interpretation of RDTs [9,10]. Clearly written instructions can add to the comprehensibility and maximize RDT kit performance [9,10]. On this basis, the World Health Organization (WHO) designed easy-to-read generic job aids [11].

During field visits in Africa, teams of the Institute of Tropical Medicine (ITM) occasionally noted shortcomings in RDT kit boxes, content and instructions. In addition, part of the interpretation errors that were observed during a recent external quality assessment (EQA) on RDTs were shown to be related to errors in the information inserts of the RDT kits used [4]. Inspection of these information inserts also revealed a large variety in layout and readability, as well as variations in the adequacy of labelling of RDT boxes and devices.

### Objectives of the present study

In view of the observations above, it was decided (i) to assess malaria RDT kits for adequate and correct design, construction and labelling of boxes and components, and (ii) to study the readability and accuracy of their information inserts.

## Methods

### Selection of RDT kits

Malaria RDTs marketed as devices consisting of cassettes, cardboard boxes and hybrids (nitrocellulose strips to be dipped into plastic wells) were selected. They were checked for the presence of the CE label and evidence of good manufacturing practice (GMP) based on their inclusion in the WHO lists of RDT manufacturers and distributors complying with ISO13485:2003 or US FDA 21 CFR 820 production norms [12].

As this study was not intended to score RDTs individually, it was decided not to display the RDT brand and kit names, in line with previous comparative studies assessing RDTs [3-5,13].

### Criteria used for RDT kit assessment and procedure

For packaging, design and labelling, assessment criteria were compiled from requirements listed in regulatory documents such as the Directive 98/79/EC and the European Community (EC) as well as relevant WHO documents [14-17]. Criteria for information inserts and device design from studies on end-users' performance and RDT instructions were pooled [5,7,9,10,18-24]. Inadequacies were defined as listed in Table 1.

**Table 1:** Number of RDTs (n = 42) with inadequacies in malaria RDT boxes, device packages, devices, buffer vials and package inserts*

Items considered to be inadequate		Number (%)
**Box: construction and design**		
	Materials: plastic bag or simple cardboard (not humidity-resistant)	9 (21.4)
	No labels, no printed information or labels not humidity-resistant	6 (14.3)
	Differences in name on device packaging, device, buffer and information insert	27 (64.3)
**Box: information displayed**		
	No EC-REP mentioned on CE labelled RDTs, although required (n = 25)	3 (12.0)
	RDT kit's name nor additional information refer to intended use	3 (7.1)
	RDT kit's name incorrectly refers to *P. vivax *instead of non-*falciparum *species (n = 29)	4 (13.8)
	Kit components not displayed	26 (62%)
	Essential information lacking: expiry date, numbers of tests included, storage conditions	12 (28.6)
**Kit contents:**		
	Capillary sampling system (lancet and alcohol swap) not included or not optionally included	24 (57%)
	Blood transfer system (capillary, pipette or tube) not included	3 (7.1%)
**Device package and content: construction and design**		
	Material not humidity-resistant	4 (9.5)
	No desiccant or desiccant without saturation indicator	18 (42.9)
**Device package and content: information displayed**		
	Essential information lacking: expiry date, lot number, test kit name	9 (21.4)
	No warning label "do not swallow" on desiccant	6 (14.3)
**Device: construction and design**		
	Space for sample identification too small or not writable with standard pen (felt pen needed)	40 (95.2)
	No or incomplete RDT name on the device	29 (69.0)
	No reading label or simultaneous presence two reading labels consisting of symbols only	6 (14.3)
**Buffer: construction and design**		
	Buffer vial not leak proof	2 (4.8)
	Label does not stick well to the vial, prints are not humidity-resistant (n = 40)	16 (40.0)
**Buffer: information displayed**		
	Essential information lacking: expiry date, lot number, storage conditions, correct RDT kit's name (n = 41)	24 (58.5)
	No instructions included on how to pierce the buffer vial dropper (n = 15)	5 (33.3)
**Package insert: information**		
	Absence of date of release or version number	20 (47.6)
**Package insert: content**		
	Identity of target antigens not clearly mentioned	2 (4.8)
	No referral to biosafety precautions (gloves, safe waste disposal, etc.)	18 (42.9)
	Major differences between depicted and real device (n = 40)	8 (20.0)
	Use of figures with unrealistic colours (*e.g*. control and test lines depicted as green)	4 (9.5)
	No data on test characteristics (sensitivity, specificity)	8 (19.0)

* Total number of RDT kits = 42 unless otherwise stated.

### RDT kit package, device package, device and buffer vial

The RDT kit packages were assessed for type (box versus plastic bag), material (simple and plasticized cardboard) and the presence and quality of the printed information. Information displayed on the package considered as essential included the RDT kits and manufacturer's names, expiry date, number of tests included, storage requirements and a reminder to read the instructions before use. A referral to the intended use of the RDT kit was looked for, either by the RDT kit name or by an additional text. The expiry date mentioned on the box was matched with those of the other RDT kit components. For CE labelled RDT kits produced by companies outside the European Economic Area, the affixing of the so-called authorized representative of the company in the EC (EC-REP) was assessed. Kits were assessed for sampling material needed included or not.

The package of the test device was checked for quality (humidity-proof material) and essential information including RDT kit name, lot number and expiry date. In addition, the desiccant was checked for composition, warning label and presence of colour indicator.

The RDT devices (cassette or cardboard housing the nitrocellulose strip) were assessed for clearness of design and construction including referral to the RDT kit's name. The space allocated for sample identification was evaluated for dimensions and ease of writing. A space of minimal 0.5 cm height and 4 cm wide was considered as adequate for handwriting of sample identification. The labelling of buffer wells, sample wells and reading windows including the places of appearance of the control and test lines (further referred to as reading label) were assessed for visibility and unambiguous interpretation.

The buffer vials were assessed for leak-proof closure, and their labels for quality of adherence and print. The information displayed on the label was assessed for the presence of RDT kit name, lot number, expiry date and storage conditions.

### RDT information insert

RDT kits were checked for the presence of an information insert and a job aids (short procedure version), of which date of release and version number were assessed.

#### Layout and figures

The figures were counted and their dimensions measured. Their total surface area was calculated and expressed as a percentage of the total surface of the information insert. The figures were assessed for their concept (pure black and white versus use of colours) and conformity with the real devices.

#### Typography

The font size of the predominant letter type used (excluding the bibliography section) was measured in Cicero using a typometer (Rotring-werke Riepe KG, Hamburg, Germany) as the "kp" distance from the top of the highest ascender (top of the lower case letter k) to the bottom of the lowest descender (bottom of the lower case letter p). The opening of the characters was visually assessed for the characters "c, o and a", by covering them for their lower two-thirds and checking whether they were still correctly readable (open letter type) versus read as an "o" (closed letter type). The interline spacing was assessed by measuring in Cicero with a typometer the distance between the base line of two successive rows and then subtracting the font size. Fonts of open letter types and interline spacing equal or larger than 2 are better readable compared to fonts of closed letter types and interline spacing smaller than 2, especially at larger text columns. For patient education materials and health instructions, font sizes of 12 or larger are recommended [25,26].

#### Readability level

For assessment of the readability level, the sessions about blood sampling, procedure and interpretation in the English text version were copied or retyped in Microsoft Word (Microsoft Corp., Redmond, WA, U.S.A.) and checked for correct spelling and syntax construction. Follow-up editing was performed as described elsewhere [27]. Next, the text fragments were copied into an on-line readability assessment tool, which generates different reading indices [28]. The Flesch-Kincaid grade level [29] was calculated. This grade-level expresses the U.S. grade-level equivalency of the skills required to read a particular document. For patient education materials and health related information, the recommended level is ≤ 6^th ^grade level [25,30].

#### Accuracy and relevance of information

The following items were actively looked for: description of the RDT test principle, target antigens, listing of required materials provided and not, description of sampling procedures and biosafety precautions. The RDT test procedures were studied with reference to common errors made by end-users in the field (Table 2) [5,9-11,18,19,23]. The interpretation section was assessed for the complete description of invalid results and *Plasmodium *species differentiation as well as for listing causes of false negative and false positive results. The description of test characteristics was assessed for mentioning the diagnostic accuracies related to the different *Plasmodium *species and parasite densities. Bibliographic references were checked for relevance with regard to RDT performance in general and information on the RDT kit's performance in particular.

**Table 2:** Number of RDT information inserts (n = 40) addressing critical steps in procedure and interpretation

**Items addressed in procedure section**	**Number (%)**
	
Bring the RDT device and buffer to room temperature	32 (80.0)
Check the integrity of the device package	9 (22.5)
Check expiry date	27 (67.5)
Use the device immediately after opening	28 (70.0)
Place the device on a level surface	0 (0.0)
Check the desiccant for signs of exposure to humidity	11 (27.5)
Write down sample identification	3 (7.5)
Wipe finger with alcohol	26 (65.0)
Allow the finger to dry before pricking	12 (30.0)
Hold the transfer device (loop, straw) vertical	8 (20.0)
Hold the buffer vial vertical	12 (30.0)
Do not to use another buffer than the one provided with the kit	9 (22.5)
Use an adequate light source for reading	3 (7.5)
	
	
**Items addressed in interpretation section**	**Number (%)**
	
	
All possible line combinations for invalid test results are mentioned	12 (30.0)
All possible test line combinations for positive test results are mentioned	31 (77.5)
Interpretation of a faint test line as positive is mentioned	8 (20.0)
Causes of false negative results are mentioned, in particular low parasite densities	11 (27.5)
Causes of false positive results are mentioned, *e.g*. presence of the rheumatoid factor	3 (7.5)
Persistence of HRP-2 is mentioned	19 (47.5)
To repeat the test in case of a negative RDT result and persistent suspicion of malaria is mentioned	1 (2.5)

### Assessment, data registration and statistical analysis

Two observers trained in the use of RDTs independently assessed the RDTs according to the described criteria. Discrepant observations were discussed together with the other investigators and a consensus was reached. Data were registered in an Excel sheet (Microsoft Corp., Redmond, WA, U.S.A.).

## Results

### Panel of RDT kits

For the purpose of this study, 51 RDT kits were ordered at 29 companies. Seven companies (representing nine RDT kits) did not reply despite several reminders. The final panel consisted of 42 RDT kits from 22 companies. Nearly all (39/42, 93%) RDT kit formats were cassettes, further there were one cardboard and two hybrid kits. Two RDT kits consisted of individually wrapped RDT packages containing all materials for a single test (cassette, disinfectant, lancet and buffer). They will be further referred to as "Single RDT kits". Table 3 lists the RDTs according to their evidence of GMP. Table 1 lists the number of the RDTs with inadequacies in boxes, devices, buffer vials and information inserts.

**Table 3:** Overview of the RDT kits evaluated in the present study

			**Evidence of GMP**		
			
**RDT format**	***Plasmodium *antigens targeted**	**Number**	**CE mark**	**WHO list**^†^	**Total**
					
**Two band**	HRP-2	7	4	5	7
	pan-pLDH	1	1	0	1
	Pv-pLDH	1	1	0	1
					
					
**Three band**	HRP-2, pan-pLDH	11	5	7	9
	HRP-2, aldolase^‡^	5	5*	5	5
	HRP-2, Pv-pLDH	4	1	1	2
	Pf-pLDH, pan-pLDH	6	5	6	6
					
					
**Four band**	HRP-2, Pv-pLDH, pan-pLDH	7	3	5	6

* One three-band test (HRP-2, aldolase) is FDA approved.

^† ^WHO list of ISO:13485:2003 certified manufacturers and their RDT products (list of known commercially available antigen detecting malaria RDTs) [12].

^‡ ^One of these RDTs used both aldolase and pan-pLDH as pan-malarial antigen.

### RTD kit package, device and buffer vial

#### RDT kit package

Thirty-eight RDT kits arrived as cardboard boxes; four kits arrived in plastic bags. Two of these plastic bags contained a cardboard box to be folded by the end-user, resulting in a total of 40 boxes and two plastic bags as the package of use on the bench. All but one box displayed an indication in the RDT kit's name or in the test description that the RDT kit was intended for malaria diagnosis. The two plastic bags did not display any information. A company name was listed on all the 40 boxes, but for 12 kits, it was not clearly mentioned whether this name represented the manufacturer or the distributor.

The lot number and expiry date were listed on all boxes. One of the Single RDT kits showed both lot number and expiry date on the outer box containing the single packages, but not on the single packages themselves. There were no discrepancies between the expiry dates on the RDT kit box and those of the contents except for two buffer vials with expiry dates extending those printed on the RDT kit box.

The number of tests included and a reminder to read the instructions before use were not displayed on four and ten boxes respectively. All the 40 boxes showed information on storage temperature requirements, by written text, symbols or both. Apart from a single symbol, *i.e*. a penguin expressing "do not freeze", all symbols were internationally recognized symbols complying with EN 980:2008 or FDA 2004, 21 CFR 809.10 and 21 CFR Parts 610 and 660. Capillary blood sampling systems were included in eight kits and proposed as optional in ten other kits. Blood transfer systems were missing in three kits.

#### Device packages

Four of 42 device packages were not made of humidity-resistant material. Most of the packages were easy to open by tearing a pre-cut lid of the package. However, for three packages, scissors had to be used to open the packages properly.

A desiccant was present in all but one package. Three of the 41 desiccants did not show a warning that the desiccant was harmful: two of them (from one manufacturer) were tablets looking like drug pills. Seventeen desiccants (including the two tablets) had no colour indicator of humidity saturation.

#### RDT devices

**Figure 1:**
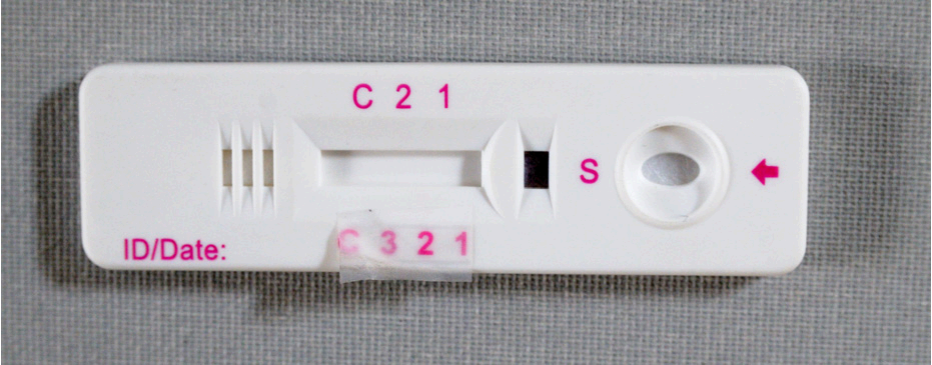
**Four-band RDT**. The allocated place for writing sample identification is too small. The grid at the left hand may be confused with a sample well. There are two different reading labels at each side of the reading window, of which the lower one is printed on a label that is not well fixed.

Space for writing was too small in 40 of the 42 devices (Figure 1). For two cassettes, a felt pen was required as a standard pen failed to mark.

**Figure 2:**
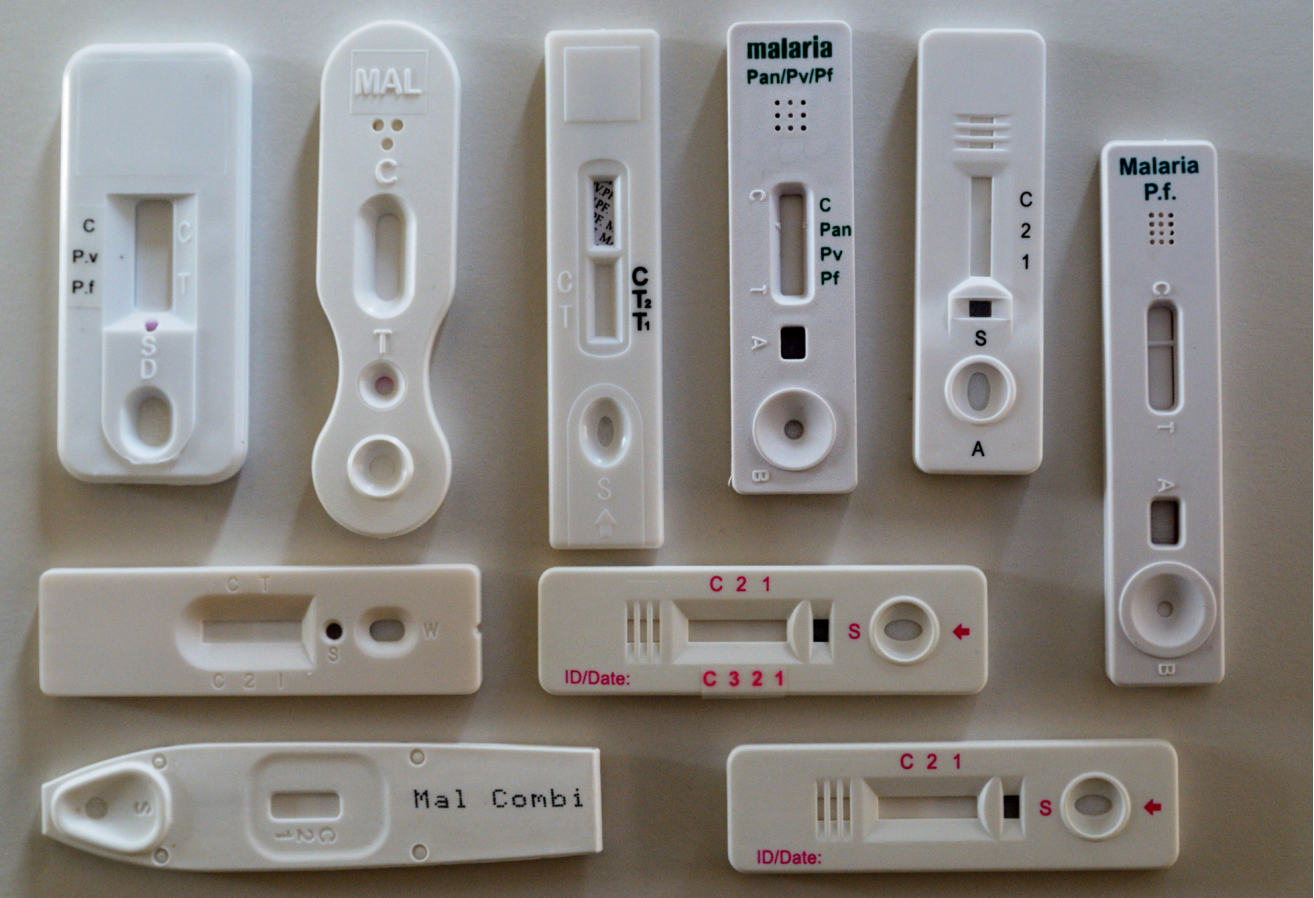
**Example of RDT cassettes**. Most of the cassettes have separated wells for sample and buffer application. There is no uniform labelling of the wells: different characters (*e.g*. "S", "A") are used randomly for the sample well, buffer well and combined sample/buffer well. The reading labels are indicated with acronyms, characters or numbers.

Most cassettes (35/39) had separate wells for sample and buffer application, four had a single well for sample and buffer application. There was no uniform labelling of the wells: for instance characters "S" and "A" were used randomly for the sample well, buffer well and combined sample/buffer well (Figure 2). Fifteen cassettes showed at the distal end a window or holes that might be confused with a sample or buffer well (Figure 1 and 2).

The reading label was indicated with acronyms (n = 20), characters or numbers. Acronyms included abbreviations such as "Pf" or "pan", they were printed on the plastic housing or on a label and were well readable. Characters such as "C" (control line) and "T" and numbers were embedded in the plastic housings and were more difficult to distinguish (Figure 2). In one cassette, characters were printed on a label, which was not well fixed (Figure 1). In 14 cassettes, two labels were displayed at either side of the reading window (Figure 2), and one three-band cassette had no reading label at all (Figure 2).

#### RDT buffer vials

The two Single kit RDTs contained a small buffer plastic ampoule in each device package, which were too small to display information. Fifteen buffer vials required clockwise tightening the vial cap to pierce the dropper vial nozzle, but for five of them, this was not mentioned in the information insert. For 13 vials, the label was not well fixed and the printed information on three of these labels was not humidity-resistant. Lot number and expiry date were not listed on five vials and storage conditions were missing on 10 vials.

### RDT information insert

The information inserts of the two Single RDT kits were not considered: one of them contained a simple job aids explaining the procedure by figures only, the other contained a shortened version of the information insert of the same RDT marketed as laboratory kit. All of the remaining 40 RDT kits contained an information insert of which seven had an additional job aids. Either version number or date of issue was missing in 11 and 13 of them; in five, both were missing.

#### Layout and figures

All 40 inserts included figures. The median number of figures per information insert was 8.5 (range 2 - 25) and figures accounted for a median surface ratio of 7.2% (range 0.4% - 33%) of the entire insert. The median size of the figures was 2.4 × 2.0 cm, the smallest and largest figure measured respectively 1.0 × 0.3 cm and 6.7 × 8.0 cm.

**Figure 3:**
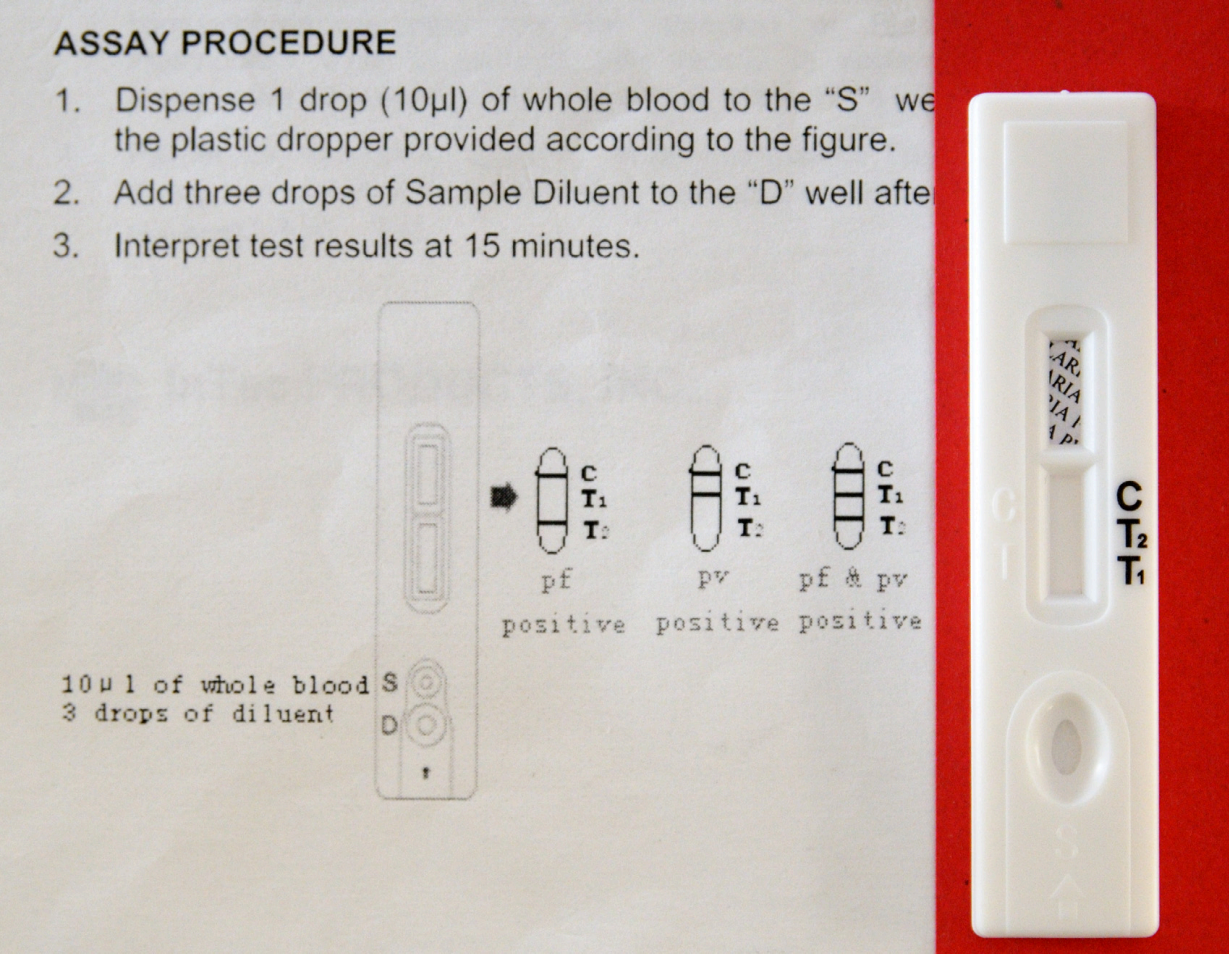
**Interpretation section of the information insert and cassette of a *Plasmodium falciparum/Plasmodium vivax *RDT**. The real device has a single sample/buffer whereas the depicted one displays separate wells. The characters used for the reading label on the illustration are inverted compared to the real device.

**Figure 4:**
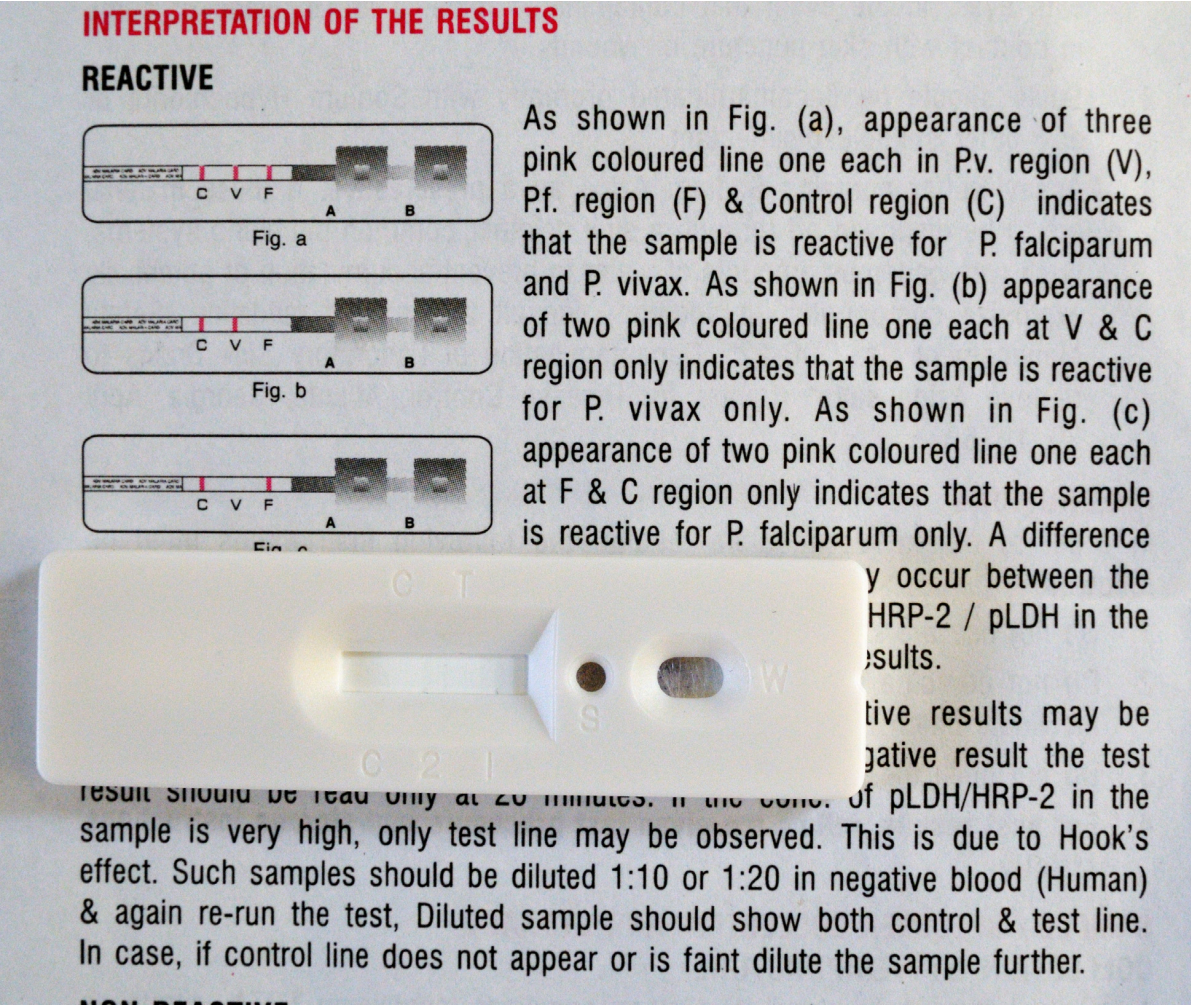
**Interpretation section of the information insert and cassette of a *P. falciparum/P. vivax *RDT**. Shape and labels of wells and reading window are different between the real and the depicted device. Characters are embedded in the plastic housing and poorly discernable. The text is correct and complete (even the prozone effect and how to deal with it) but less readable (Flesch-Kincaid grade level 9.1).

**Figure 5:**
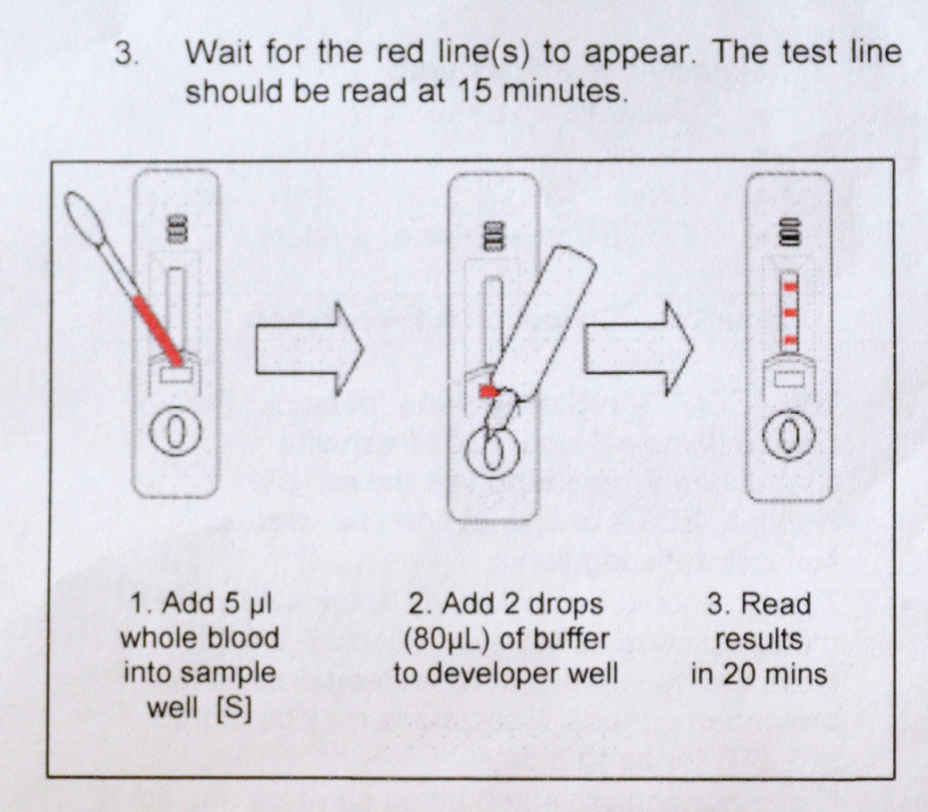
**Information insert of a three-band RDT, procedure section**. There is a discrepancy between the reading time mentioned in the text compared to that showed on the illustration.

All inserts used figures to illustrate the interpretation section. Other figures depicted blood sampling (n = 17), application of sample and buffer (n = 21) and a clock indicating the correct reading delay (n = 10). Fourteen inserts used red colour to indicate control and test lines but in four, they were pictured green or blue. Most inserts (n = 35) showed differences between depicted and real devices of which some were major, such as discrepancies between characters used for sample and buffer well identification (n = 4) and differences in labelling of the reading window (n = 5) (Figures 3 and 4). One insert mentioned a reading delay of 15 minutes, but the illustration mentioned a reading delay of 20 minutes (Figure 5).

#### Typographic features and readability

**Figure 6:**
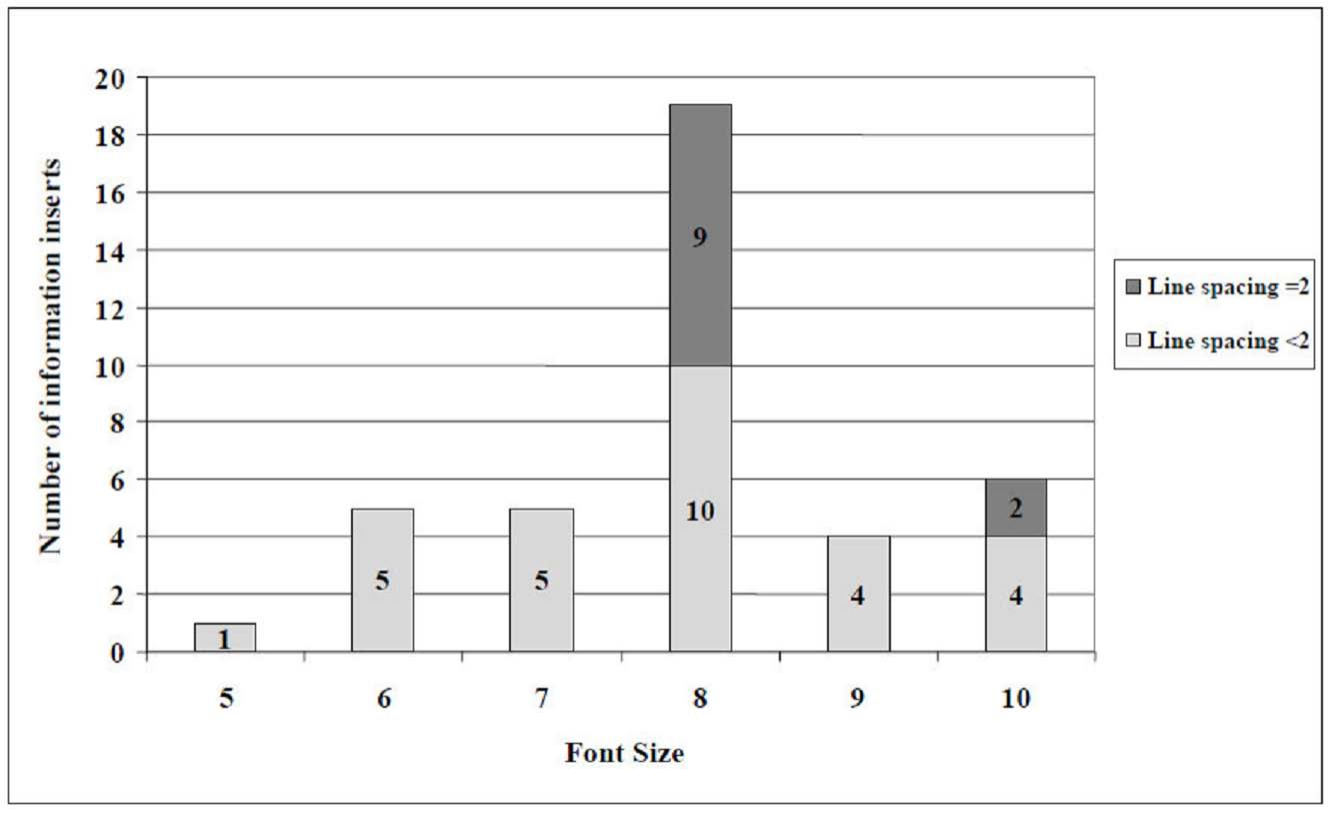
**Typographic features of RDT information inserts (n = 40): font sizes and line spacing**.

**Figure 7:**
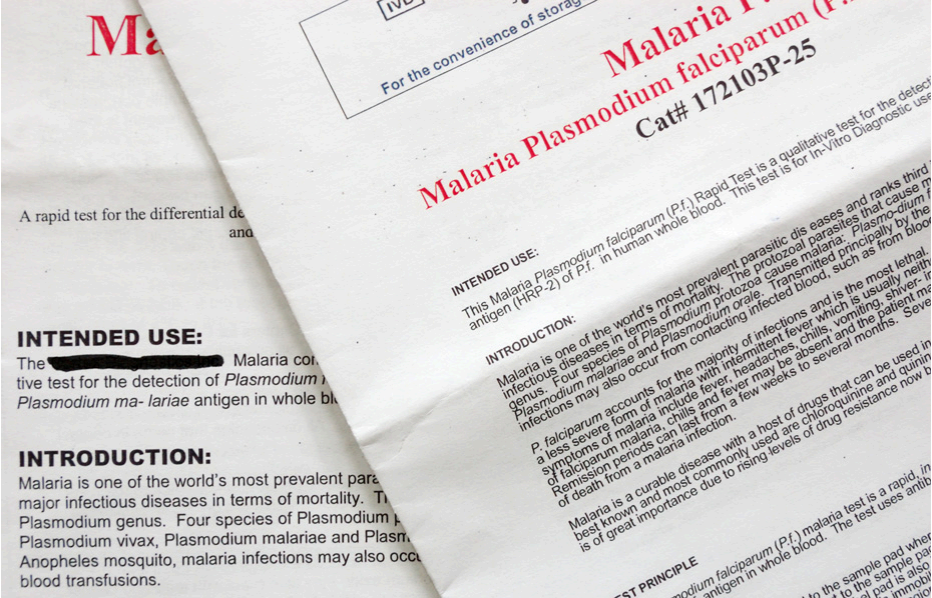
**Example of typography used in RDT information inserts: the package insert on the top is user-unfriendly (font size 6, line spacing 0,5, close letter type, average number of words per line 21, Flesch-Kincaid grade 9,5)**. The package insert on the background, from the same company but for another RDT uses a better typography (font size 8, line spacing 2, open letter type, average number of words per line 14), but the readability is still elevated (Flesch-Kincaid grade 9,8).

**Figure 8:**
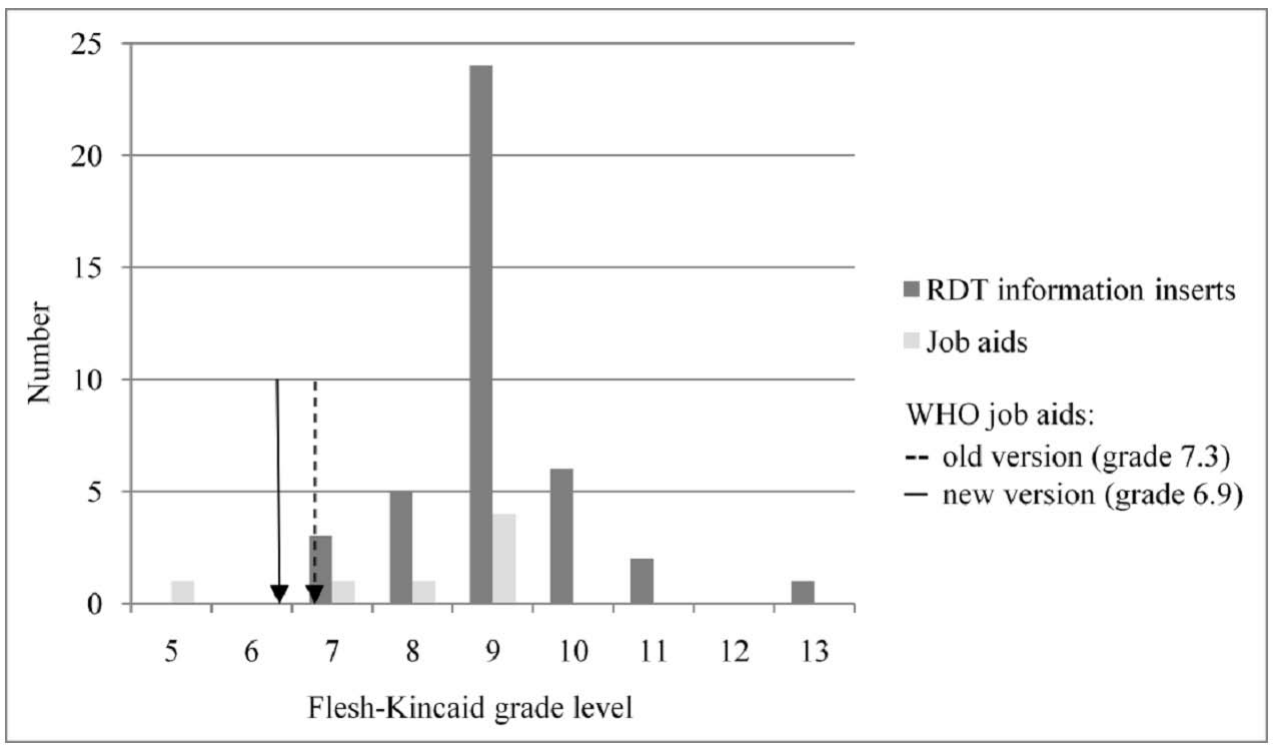
**Readability of the RDT information inserts (n = 40) and job aids (n = 7) expressed as Flesch-Kincaid grade level**.

**Figure 9:**
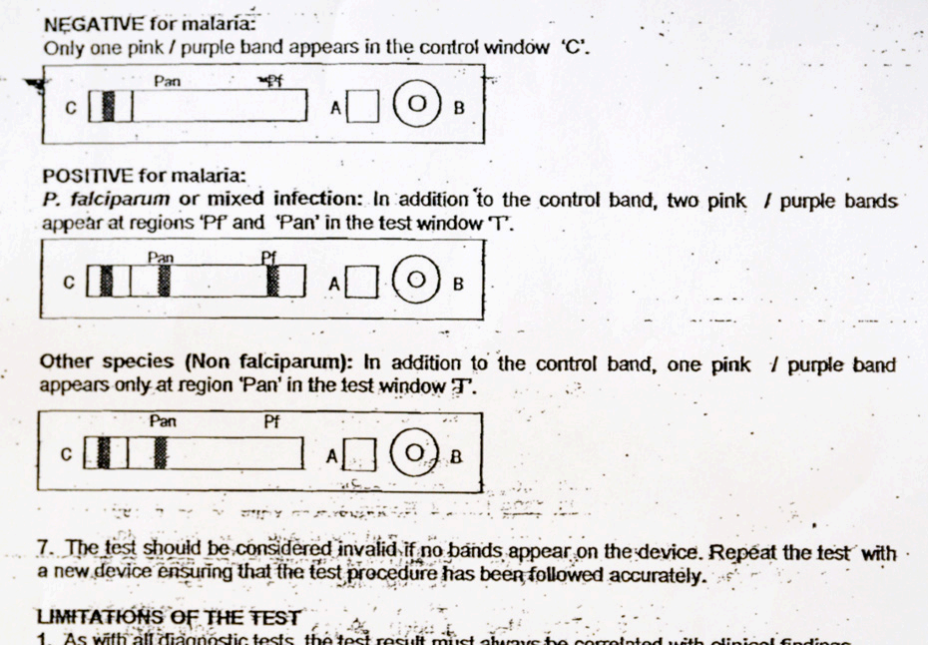
**Interpretation section of the information insert of a three-band *P. falciparum*/pan-species RDT**. For invalid tests, only the absence of all lines is mentioned, not the possibility of a visible test line without a control line. The invalid results are not depicted. The print quality is poor, no colours are used for the control and test lines.

Figure 6 displays the font size and line spacing of the information inserts. Median font size was 8 ± 1.3, none of them exceeded 10. User-unfriendly typographic features included combinations of font sizes of eight or smaller with a closed letter type (n = 10) or with line spacing lower than two and more than 12 words per line (n = 14) (Figure 7). Median readability level was grade 8.9 (range 7.1 - 12.9) and 18 and four of the inserts' readability levels were above grade 9 and 10 respectively (Figure 8). Readability levels of the job aids were also high (median 8.5, range 5.1 - 9.4), and six out of seven exceeded the readability level of the most recent WHO job aids. Five inserts showed prints of very poor quality hindering reading of the text (Figure 9).

#### Accuracy and relevance of information

The RDT kit's principle was described in all 40 information inserts. All but two inserts mentioned the materials provided in the RDT kit, and seven inserts provided a complete list of the materials required.

All RDT kit inserts mentioned the required specimen (in all cases both capillary and venous blood), all but one mentioned the anticoagulant to be used. Capillary blood sampling through finger prick was described in 35 inserts, of which one also added sampling by venipuncture. By contrast, the heel prick was not described in any information insert.

**Figure 10:**
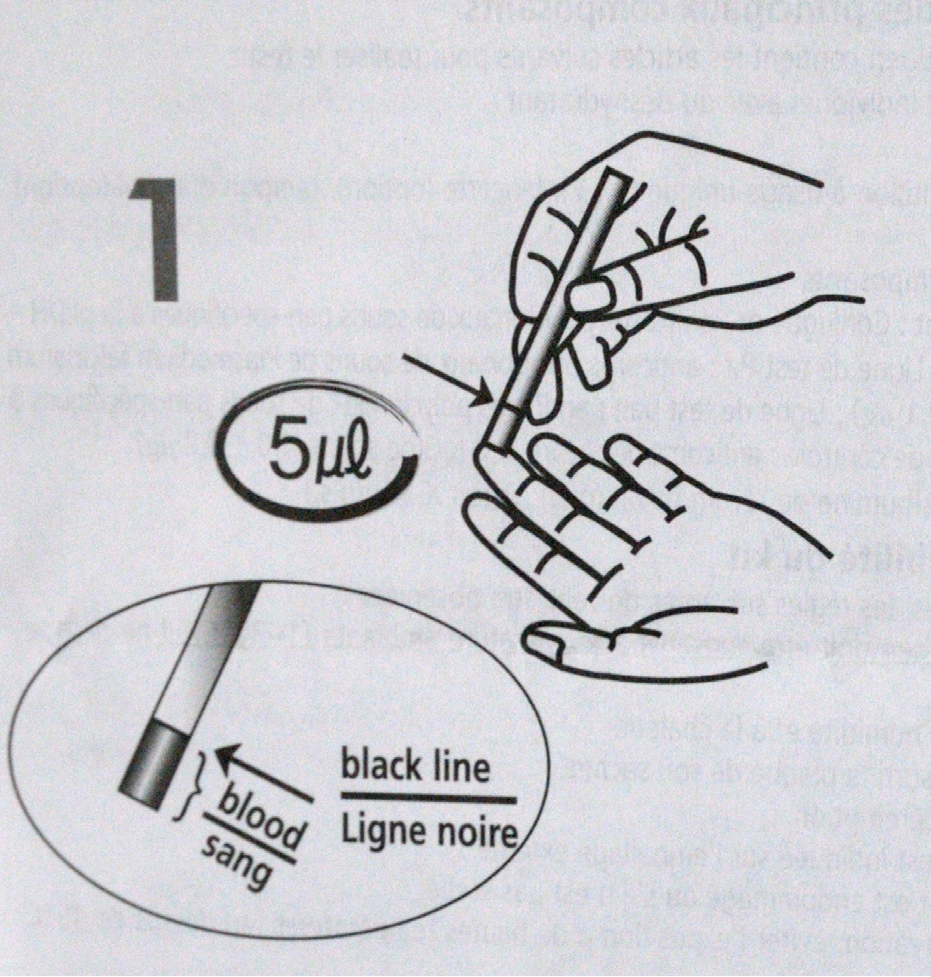
**Illustration depicting sampling of capillary blood**. The health care worker's hand is depicted without gloves. The simultaneous presence of English and French text may be difficult for a non-experienced reader.

Biosafety precautions included the use of gloves (depicted or mentioned in 21 inserts, Figure 10) and safe waste disposal (addressed in 16 inserts), but 18 inserts did not mention any information on biosafety.

From Table 2 it is clear that a number of critical steps in RDT procedures were addressed by only part of the RDT inserts. Among them, there were relevant steps such as writing down sample identification, correct positioning of the transfer and buffer vial and the need for an adequate light source.

The complete array of all control and test line combinations was listed by only nine inserts. Fourteen inserts mentioned the absence of all lines as an invalid result but not the presence of a test line in the absence of a control line (Figure 9). In addition, errors in the interpretation of test lines were observed. For instance, the combination of a Pf-specific and a pan-specific test line was interpreted as *P. falciparum *without mentioning the possibility of a mixed infection (12/34 three- and four-band RDTs). Likewise, the combination of a Pv-specific and a pan-specific test line in case of a four-band RDT was interpreted as a *Plasmodium vivax *infection without mentioning the possibility of a mixed infection with *Plasmodium ovale *and *Plasmodium malariae *(6/7 RDTs). In addition, a visible pan-pLDH line was interpreted as a *P. vivax *infection (instead of non-*falciparum *species) in two inserts.

Few inserts mentioned causes of false positive and false negative results (Table 2). One insert recommended to repeat the test in case of a negative RDT result and persistent suspicion of malaria, another warned about the prozone effect as a cause of a false negative result (Figure 4). RDT test results during treatment follow-up were addressed in 13 inserts, but the information listed in nine inserts was presented in a scattered way and only one insert clearly mentioned that HRP-2 persistence does not indicate a failed therapeutic response.

#### RDT test characteristics

Eight inserts did not provide information on sensitivity or specificity. Diagnostic characteristics were mostly expressed for *P. falciparum *and *P. vivax *(n = 31 and n = 22 respectively), only one insert mentioned test characteristics for *P. ovale *and *P. malariae*. Sensitivity for *P. falciparum *was expressed by parasite density range in 10 inserts.

### Bibliography cited in the information inserts

In total, 45 different references were used in the bibliography of the information inserts. One third of them referred to the original description of the target antigens, another 12 referred to general information on malaria and its diagnosis. Thirteen inserts cited evaluation studies of RDTs, but only three RDT kits referred to product-related studies. Two panels of identical references were shared by nine and eight inserts respectively.

#### RDT kits' names

Inconsistencies in the RDT kit names and referrals to target antigens were observed. For instance, four RDT kits had names referring to *P. vivax *although they used a pan-species *Plasmodium *antigen. Two other inserts did not mention the identity of the pan-species antigen (aldolase versus pan-pLDH). Five RDT kits from one manufacturer were supplied in identical boxes, carrying the same names and identical prints. Furthermore, there were slight but numerous differences between names as displayed on boxes versus those noted on device packages (eight had no brand name affixed), devices (15 differences) and information inserts (eight differences). Similar observations were made for buffer vials: five vials displayed only the manufacturer's name and one vial did not show brand nor manufacturer's name.

#### RDT kits' duplicates

During assessment of the RDT kits, apparent similarities between different RDT brands were observed. These similarities concerned, amongst others, design and shape of the device and content and layout of the information insert (*e.g*. numbers of samples used for calculation of test characteristics). In that way, six products were assumed to represent a common design and production platform for 16 different RDT brands.

#### Relation with CE marking and WHO listing

**Figure 11:**
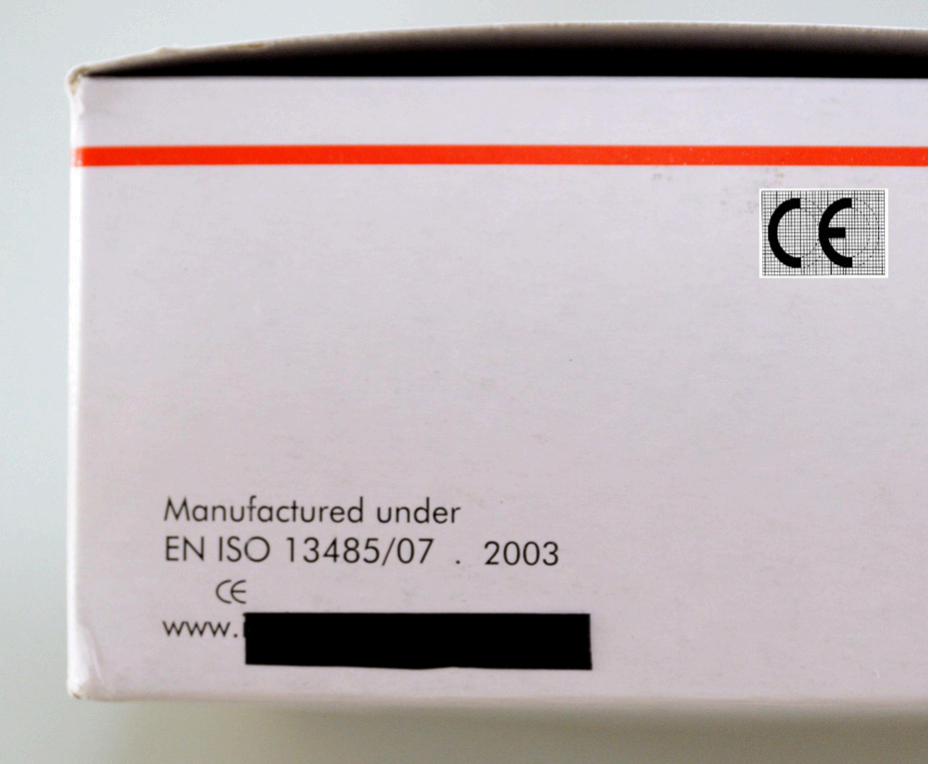
**CE-label displayed on a RDT kit box**. The shape and relative dimensions of the characters do not comply with the requirements as mentioned in the EC Directive 98/79 (depicted in the insert, upper right corner). There is no authorized representative (EC-REP) affixed, although required.

Overall, RDTs with evidence of GMP (n = 35) scored better compared to those without (n = 5), although inadequacies, errors and omissions were observed between both groups. Three CE labelled RDT kits produced outside European Economic Area (EEA) had no EC-REP indicated neither on the box nor in the information insert. For these kits, the CE symbol as displayed on the package had not the shape and relative dimensions of the 98/79/EC directive (Figure 11). Six of the eight RDT kits that did not mention data on sensitivity and specificity were CE labelled.

## Discussion

Previous studies demonstrated that RDT manufacturers' instructions are insufficient to ensure accurate test performance by community health care workers, and well-designed instructions such as the WHO generic job aids have proven to increase performance [9,10,19,31]. In practice however, such job aids still need to be adapted to the particular RDT brand used on site and, depending on the chains of supply, different RDT brands and versions may be available. For market exploration and choice of RDTs, laboratory managers will orient to RDT kits' names and labelling. Clear design and labelling of RDT kit components will contribute to correct storage and use; and laboratory staff will rely on RDT inserts for background information, adaptations of the generic procedures, interpretation and trouble-shooting.

The design of the present study has its limitations. For instance, mainly RDTs marketed as cassettes were considered. However, cassettes are the mostly used platform and preferred by end-users over the strip format [9]. Besides, only a part of the marketed brands was evaluated; however, this evaluation studied about half of the 80 brands worldwide-marketed [2] including those frequently used in endemic and non-endemic settings.

With regard to the assessment of the information inserts, it should further be noted that the Flesch-Kincaid label (as discussed below) is only a proxy measure of readability. In addition, the layout of the inserts was assessed for typography but not for other features such as adequate use of headings, bullets, boldfacing, and amount of white space [32,33]. Finally, although RDTs were presently assessed against compiled criteria based on relevant documents, they were not evaluated by end-users in a real-life setting. On the other hand, as far as known, this is the first time that in vitro diagnostic medical devices (IVDs) were assessed for these characteristics.

### Problems in design and labelling of the RDT kits' components

Despite these limitations, much information was generated on the quality and appropriateness of RDT packaging, labels and inserts. Apart from two exceptions (the plastic bags), boxes were well labelled, but shortcomings in labelling of device packages and buffer vials were more numerous. The absence of blood sampling and transfer system which was observed in nearly a quarter of RDT kits may create logistical problems when used in field settings. The problems in device design were of most concern. Characters indicating wells and reading labels that were embedded in the plastic cassette housings are difficult to distinguish and the simultaneous presence of two reading labels may cause confusion. Standardized and unequivocal characters or acronyms should be used for designating wells and reading labels and clear labelling with contrasting print should be ensured.

Some design issues were not compiled from previous studies but originated from ITM observations. Some of them may look trivial but have consequences in daily practice. For instance, device packages without pre-cut lids require scissors to open, which is neither safe nor practical in busy and remote settings. The space allocated for sample identification on most cassettes was large enough for writing down a sample number but not a patient name: this may meet the requirements in computerized settings but not those in a non-computerized field setting, where full names are written as recommended by WHO job aids instructions [16]. Likewise, it is evident that use of a felt pen for writing down sample identification is inappropriate for a field setting. Another example was the clockwise tightening of the buffer vial's cap for piercing the dropper bottle nozzle: ITM teams observed that laboratory staff unaware of this procedure simply cut off the distal end from the nozzle. The resulting opening tended to be very wide resulting in a too large volume of buffer added to each test and early emptying of the buffer vial. The RDT cassettes that remained without buffer were run with other buffers or with injection water, at the risk of causing false positive results [5].

### Readability level and typography of the information insert

The Flesch-Kincaid grade level used in the present study has been demonstrated to be reliable and valid and is frequently used in health care issues such as consumer medical information (CMI) and patient education materials [27,30]. Its use in the present context should be interpreted with caution. Like any other reading formula, the Flesch-Kincaid readability tool assesses text structure but does not take into account the content. It further refers only to US grade levels and applies to English language. Other factors such as motivation and previous experience may influence comprehension, and linguistic and cultural issues may interfere [33]. Despite these limitations, it is of note that readability levels of all inserts exceeded the 6^th ^grade level, while the recommended level for health related information is equal or lower than this level [27,30]. The readability level of the job aids scored slightly better, but still higher compared to the WHO generic job aids. Too elevated reading levels have been consistently demonstrated in CMI documents such as those of home pregnancy tests, blood glucose monitoring and home blood pressure monitor equipment as well as in patient education brochures [25-27,32,34,35]. It should also be taken into account that end-users in endemic settings are likely to be non-native speakers of the language of the information insert (*e.g*. English, French, Portuguese), and may operate in stressful situations such as environmental disasters and war [36,37] which decrease actual reading levels [38]. Apart from the bad quality prints and small figures, the observed user-unfriendly typographic features may add to the decreased readability of the inserts, which were consistent with those documented for CMI materials [25,30,32,34,35].

### Content of the information insert

The lack of referral to biosafety procedures in nearly half of the inserts was striking and unacceptable, in particular because this is clearly mentioned in the WHO generic job aids [11]. Likewise were the differences between depicted and real devices and the use of non-realistic colours for depicting test lines which do not comply with WHO recommendations [16]. Numbers and sizes of illustrations did not comply with the established standards for patient education materials and CMI [25,35,39].

The shortcomings in the RDT test interpretation session were in line with observations made during a recent external quality assessment on RDTs in a non-endemic setting. During that session, not reporting a mixed infection in case of the simultaneous presence of *P. falciparum*- and pan-specific test lines was demonstrated to be linked to the information inserts of the RDT kits used [18]. Although extremely rare in the experience at ITM, the presence of a visible test line in the absence of a control line points to invalid test results and should be added to the spectrum of possible line combinations, preferably with a picture. In addition, the persistence of HRP-2 after successful treatment and the production of pLDH by gametocytes should be clearly mentioned.

The poor description of diagnostic characteristics in the information inserts was another concern. Although current directives and recommendations do not specify details about origin, numbers and statistical validity of these test characteristics, manufacturers should be encouraged to provide as detailed and sound data as possible, including data on sensitivity in relation to parasite density and *Plasmodium *species. The low diagnostic sensitivity for *P. malariae *and *P. ovale *is well known [40]: few studies have included enough samples to provide reliable data for both species. Those that did mostly found a poor sensitivity, in particular for *P. malariae *[40-44]. In the absence of a thorough evaluation for both species, one could consider adding a statement mentioning the low overall diagnostic sensitivity for both species to the information insert, in order to avoid unrealistic expectations by the end-user relying on the pan-*Plasmodium *species nature of the targeted antigen [40].

The cited bibliography mainly referred to the original papers on the description of the antigens or general RDT evaluations. In addition to references addressing the RDT kit itself, references to one or more of the recent reviews on RDTs or WHO/FIND documents could be added, as they contain relevant information on the use and limitations of RDTs.

### Names and duplicates of RDTs, relation to GMP and CE labelling

Among the inadequacies, erratic and inconsistent names were a frequent finding: they ranged from minor differences in RDT brand names as displayed on boxes, devices and their packages and information inserts to brand names suggesting *P. vivax *despite using a pan-pLDH target. In addition, shortcomings with regard to clear specification of target antigens were noted. For a laboratory manager finding his way among many other diagnostics and supplies, it is essential to get a quick and reliable idea about the intended use and the target antigens of RDTs. An unequivocal code for naming and short test descriptions should be considered, with mentioning of the (abbreviated) antigens as a requisite (*e.g*. Pf-pLDH, HRP-2 etc.).

With regard to the presumed RDT kit duplicates (kits presenting with similar presentation suggesting a shared design and production), it should be noted that WHO and FIND recognize this phenomenon [45]. WHO defines so-called "re-branded" products as products manufactured under identical conditions at the same manufacturing site as the original product, but labelled with a different product name and identifier. WHO encourages, in such case, joint application for the prequalification program or test evaluations [45-47]. The CE recognizes also the "re-branding" for commercial interests. The coexistence of multiple names for the same product however may create difficulties for instance in retrieving published information on test evaluations and may add to the complexity of post marketing surveillance, including traceability in case of batch recalls. To prevent these problems, the requirement of "re-branded" RDT kits to mention the original manufacturer should be considered. In addition, any RDT kit label (whether original or re-branded) should clearly distinguish the names of the manufacturer from that of the local distributor.

Shortcomings and errors were observed among CE-labelled and WHO-listed RDTs. Not affixing the EC-REP when required and not mentioning information on RDT test characteristics do not conform the 98/79/EC Directive [48]. It should further be noted that in case of malaria RDTs, the CE-label by itself is not a guarantee for intrinsic quality of performance. The 98/79/EC Directive includes the "Annex II", which lists diagnostics for which market release of any new lot has to be preceded by testing and approval by a competent authority, the so-called notified body. For diagnostics that are not listed in the "Annex II" (such as malaria RDTs), such testing and authorization are not required. Acquisition of the CE-label for these diagnostics is a purely administrative process, in which the manufacturer himself draws up the EC declaration of conformity. Unfortunately, the majority of laboratory and medical staff are unaware of this procedure. This can create a sense of "over-confidence" in CE-labelled products, based on the perception of quality associated with European labels. The inclusion of RDTs for malaria and other tropical diseases in the "Annex II" could represent a significant support for countries with weak regulatory overview.

### What can be done to improve the quality of RDT package and information inserts?

Many shortcomings such as incomplete and incorrect labelling of boxes, device packages, cassettes and buffer vials can be easily remediated at minimal costs. End-users and manufactures should reach consensus on uniform codes for labelling wells and reading windows and gradually reinforce the requirements for inserts and packaging. Generic recommendations as how to layout and how to appropriately design information inserts as well as use of figures can be found in the literature on CMI [25,35,39], RDT specific guidelines have been issued by the WHO [16]. Readability, cultural and linguistic backgrounds should be taken into account [23], and all texts and figures should be assessed for appropriateness and comprehension among the targeted end-users [10,49]. For the content of RDTs, the reference documents that were used to compile Tables 1 and 2 can give guidance. Examples of such checklists are added as additional files (Additional file 1 Table S1 and Additional file 2 Table S2). The information inserts should highlight key points in performance and interpretation in order to reduce the likelihood of potential errors.

**Figure 12:**
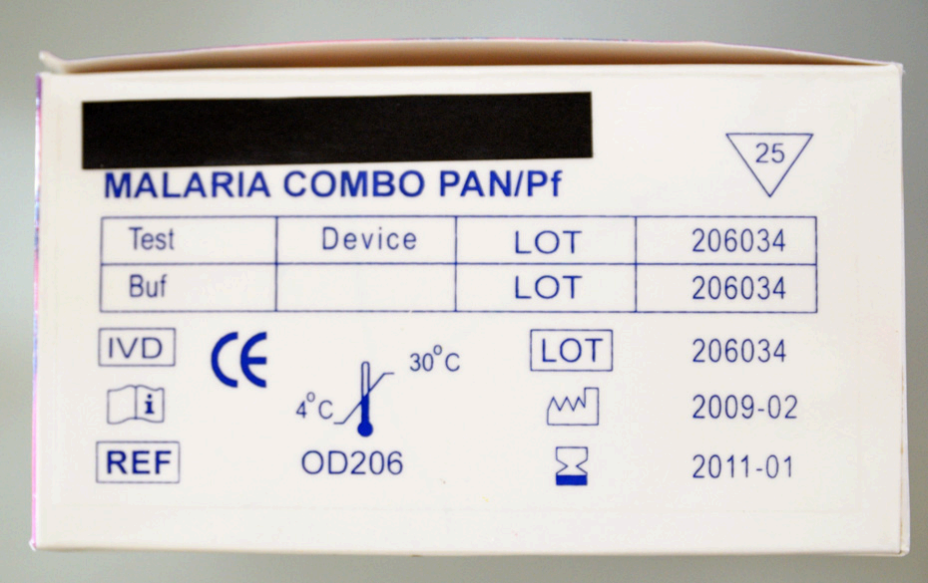
**Example of the lateral side of a RDT box: all essential information is printed on a single side of the box, contributing to clear storage**.

With regard to user-friendliness and adequacy of RDT presentation and instructions, interesting features not listed on the compiled criteria were noted. For instance, some device packages carried short instructions for use based upon the generic WHO job aids [16], witch may increase the test performance [9,10,19,31]. The availability of job aids for the different brands on the company websites in an adaptable text format may help Malaria National Programs to translate it in the end-user language and to adapt it on the local context. In addition, some RDT kits provided a glossary with explanation of the affixed symbols, which may help in their comprehension and acquisition and another five had all essential information printed on a single (lateral) side of the box, contributing to easy storage (Figure 12). Another asset was the presence of more than one buffer vial per RDT box, as shortage and replacement of buffer vials is a common problem in resource limited settings [5]. Likewise, the above described observations and corrective measures could be extended to other IVDs, such as human immunodeficiency virus RDTs.

Of course, it should be noted that adequate packages and information inserts by themselves are not a guarantee for competent use of RDTs. Simply distributing the RDTs and instructions does not work, and RDT instructions on their own will not change professional behaviours [20]. Thorough training and performance monitoring are needed for correct performance [16].

## Conclusion

In conclusion, malaria RDTs showed shortcomings with regard to quality of construction, design and labelling of boxes, device packages, devices and buffers. Information inserts were difficult to read and lacked relevant information. Particular problems were observed in the consistency and appropriateness of RDT brand names and in the referral to the antigens used. In general, CE-labelled and WHO-listed RDTs scored better compared to those without but inadequacies were observed among these RDTs. Addressing the quality of RTD package and information inserts in evaluation programs such as the WHO/FIND products testing program could stimulate the manufacturers to remediate these shortcomings. Likewise, inclusion of malaria RDTs in the "Annex" II of the 98/79/EC directive might represent a powerful support from the European Community towards the quality of *in-vitro *diagnostics in tropical countries.

## List of abbreviations

CE: Conformité Européenne; CMI: Consumer medical information; EC-REP: European Authorized Representative; EEA: European Economic Area; FDA: Food and drug administration; FIND: Foundation for Innovative New Diagnostics; FHML: Faculty of Health, Medicine and Life Sciences; GMP: Good manufacturing practice; HRP-2: Histidine-rich protein 2; ISO: International organization for standardization; ITM: Institute of Tropical Medicine; IVDs: *in vitro *diagnostic medical devices; *P: Plasmodium*; pan-pLDH: pan *Plasmodium*-specific parasite lactate dehydrogenase; Pf-pLDH: *Plasmodium falciparum*-specific parasite lactate dehydrogenase; pLDH: parasite lactate dehydrogenase; Pv-pLDH: *Plasmodium vivax*-specific parasite lactate dehydrogenase; RDT(s): Rapid diagnostic test(s); WHO: World Health Organization.

## Competing interests

The authors declare that they have no competing interests.

## Authors' contributions

PG and JM equally contributed to the present study and share first authorship. PG JM and JJ designed the study protocol, RR made substantial contributions to the concept and design of the study. PG, JM and VH carried out the test evaluations. PG, JM, CB and JJ analysed and interpret the results and drafted the manuscript. All authors critically reviewed the manuscript and approved the final manuscript.

## Supplementary Material

Additional file 1 **Example of operational checklist for packaging, labelling and instructions of RDTs**. Click here for file

Additional file 2 **Example of a checklist for the content of the information inserts**. Click here for file
