# Supplementary material for: Malaria rapid diagnostic kits: quality of packaging, design and labelling of boxes and components and readability and accuracy of information inserts
Source: Malar J. 2011 Feb 13;10:39. doi: 10.1186/1475-2875-10-39 (PMC3045995; doi:10.1186/1475-2875-10-39)
Supplement: Additional file 1 — Example of operational checklist for packaging, labelling and instructions of RDTs. [file 1475-2875-10-39-S1.DOC]

**Additional file 1:**

**Table S1:** Example of operational checklist for packaging, labelling and instructions of RDTs

| **Items to be checked** | | | |  |  |
| --- | --- | --- | --- | --- | --- |
|  |  |  |  |  |  |
| **Box: construction and design** | | | |  |  |
|  | Is the box construction humidity-resistant? | | |  |  |
|  | Are the labels on the box humidity-resistant? | | |  |  |
|  | Are names used on device packaging, device, buffer and information insert identical? | | |  |  |
| **Box: information displayed** | | | |  |  |
|  | Is the EC-REP mentioned on CE labelled RDTs when required? | | |  |  |
|  | Is there a reference to the intended use in the RDT kit's name or in additional information? | | |  |  |
|  | Is there correct reference to the targeted species (*P. vivax,* non-*falciparum* species) in the RDT kit's name? | | |  |  |
|  | Is there a list of the RDT kit’s components included in the box displayed? | | |  |  |
|  | Is all essential information present: expiry date, numbers of tests included, storage conditions? | | |  |  |
| **Kit contents:** | | | |  |  |
|  | Is a capillary blood sampling system (lancet and alcohol swab) included? | | |  |  |
|  | Is a blood transfer system (capillary, pipette or tube) included? | | |  |  |
| **Device package and content: construction and design** | | | |  |  |
|  | Is the material of the device package humidity-resistant? | | |  |  |
|  | Is it a desiccant with saturation indicator? | | |  |  |
| **Device package and content: information displayed** | | | |  |  |
|  | Is essential information present: expiry date, lot number, test kit name? | | |  |  |
|  | Is there a warning label "do not swallow" on the desiccant? | | |  |  |
| **Device: construction and design** | | | |  |  |
|  | Is the space for sample identification large enough for writing sample identification and writable with standard pen? | | |  |  |
|  | Is the complete RDT name write on the device? | | |  |  |
|  | Is it a single reading label consisting of acronyms referred to the target (*e.g.* “Pf”, “Pan”)? | | |  |  |
| **Buffer vial: construction and design** | | | |  |  |
|  | Is the buffer vial leak proof? | | |  |  |
|  | Is the label well fixed to the vial and humidity-resistant? | | |  |  |
| **Buffe vialr: information displayed** | | | |  |  |
|  | Is essential information present: expiry date, lot number, storage conditions, correct RDT kit's name? | | |  |  |
|  | Does the package insert instructions mention on how to pierce the buffer vial dropper? | | |  |  |
| **Package insert: information** | | | |  |  |
|  | Are date of release and version number present? | | |  |  |
| **Package insert: content** | | | |  |  |
|  | Is the identity of target antigens clearly mentioned? | | |  |  |
|  | Is there referral to biosafety precautions (gloves, safe waste disposal, etc.)? | | |  |  |
|  | Do the illustrations of the devices match with the real device? | | |  |  |
|  | Are realistic colours used for the illustrations (*e.g.* control and test lines depicted as red/purple)? | | |  |  |
|  | Are data on test characteristics presented (sensitivity, specificity)? | | |  |  |
