# Supplementary material for: Malaria rapid diagnostic kits: quality of packaging, design and labelling of boxes and components and readability and accuracy of information inserts
Source: Malar J. 2011 Feb 13;10:39. doi: 10.1186/1475-2875-10-39 (PMC3045995; doi:10.1186/1475-2875-10-39)
Supplement: Additional file 2 — Example of a checklist for the content of the information inserts. [file 1475-2875-10-39-S2.DOC]

**Additional file 2:**

**Table S2:** Example of a checklist for the content of the information inserts

|  |  |  |
| --- | --- | --- |
| **Items addressed in procedure section** |  |  |
|  |  |  |
| Bring the RDT device and buffer to room temperature |  |  |
| Check the integrity of the device package |  |  |
| Check expiry date |  |  |
| Use the device immediately after opening |  |  |
| Place the device on a level surface |  |  |
| Check the desiccant for signs of exposure to humidity |  |  |
| Write down sample identification |  |  |
| Wipe finger with alcohol |  |  |
| Allow the finger to dry before pricking |  |  |
| Hold the transfer device (loop, straw) vertical |  |  |
| Hold the buffer vial vertical |  |  |
| Do not to use another buffer than the one provided with the kit |  |  |
| Use an adequate light source for reading |  |  |
|  |  |  |
| **Items addressed in interpretation section** |  |  |
|  |  |  |
| All possible line combinations for invalid test results are mentioned |  |  |
| All possible test line combinations for positive test results are mentioned |  |  |
| Interpretation of a faint test line as positive is mentioned |  |  |
| Causes of false negative results are mentioned, in particular low parasite densities |  |  |
| Causes of false positive results are mentioned, *e.g.* presence of the rheumatoid factor |  |  |
| Persistence of HRP-2 is mentioned |  |  |
| To repeat the test in case of a negative RDT result and persistent suspicion of malaria is mentioned |  |  |
|  |  |  |
